# Risk Factors for Pandemic (H1N1) 2009 Virus Seroconversion among Hospital Staff, Singapore

**DOI:** 10.3201/eid1610.100516

**Published:** 2010-10

**Authors:** Mark I.C. Chen, Vernon J.M. Lee, Ian Barr, Cui Lin, Rachelle Goh, Caroline Lee, Baldev Singh, Jessie Tan, Wei-Yen Lim, Alex R. Cook, Brenda Ang, Angela Chow, Boon Huan Tan, Jimmy Loh, Robert Shaw, Kee Seng Chia, Raymond T.P. Lin, Yee Sin Leo

**Affiliations:** Author affiliations: Tan Tock Seng Hospital, Singapore (M.I.C. Chen, R. Goh, C. Lee, B. Singh, J. Tan, B. Ang, A. Chow, Y.S. Leo);; Duke-National University of Singapore Graduate Medical School, Singapore (M.I.C. Chen);; Ministry of Defence, Singapore (V.J.M. Lee);; World Health Organization Collaborating Centre for Reference and Research on Influenza, Melbourne, Victoria, Australia (I. Barr, R. Shaw);; National Public Health Laboratory, Singapore (C. Lin, R.T.P. Lin);; National University of Singapore, Singapore (W.-Y. Lim, A.R. Cook, K.S. Chia);; DSO National Laboratories, Singapore (B.H. Tan, J. Loh)

**Keywords:** Viruses, influenza, seroconversion, pandemic (H1N1) 2009, nosocomial infections, infection control, serology, vaccination, Singapore, research

## Abstract

We describe incidence and risk factors for pandemic (H1N1) 2009 virus infection in healthcare personnel during the June–September 2009 epidemic in Singapore. Personnel contributed 3 serologic samples during June–October 2009, with seroconversion defined as a >4-fold increase in hemagglutination inhibition titers to pandemic (H1N1) 2009. Of 531 participants, 35 showed evidence of seroconversion. Seroconversion rates were highest in nurses (28/290) and lowest in allied health staff (2/116). Significant risk factors on multivariate analysis were being a nurse (adjusted odds ratio [aOR] 4.5, 95% confidence interval [CI] 1.0–19.6) and working in pandemic (H1N1) 2009 isolation wards (aOR 4.5, 95% CI 1.3–15.6). Contact with pandemic (H1N1) 2009–infected colleagues (aOR 2.5, 95% CI 0.9–6.6) and larger household size (aOR 1.2, 95% CI 1.0–1.4) were of borderline significance. Our study suggests that seroconversion was associated with occupational and nonoccupational risk factors.

## Abstract 2

TOC Summary: Infection was associated with occupational and nonoccupational risk factors.

During the 2003 epidemic of severe acute respiratory syndrome (SARS), large nosocomial outbreaks of SARS occurred in several hospitals in Singapore (*1**,**2*). Since then, concerns have been raised about how emerging infections, in particular respiratory infections, could result in transmission from patients to healthcare workers and vice versa, given the high frequency and intensity of healthcare worker contacts in the hospital environment (*3*). For pandemic influenza, additional concerns exist that even mild disease might result in staff absenteeism and, subsequently, would reduce staff strength at a time of increased demand for health services (*4*).

In April 2009, a novel influenza A virus, now referred to as pandemic (H1N1) 2009 virus, emerged in the United States and Mexico and rapidly spread worldwide (*5**–**7*). Published reports on pandemic (H1N1) 2009 in healthcare workers have attributed transmission to a mixture of healthcare and nonhealthcare exposures (*8**–**10*), with varying compliance to infection control measures implicated in some transmission events (*9**,**11*). Early data from the United States suggest that healthcare workers were not overrepresented among case reports of pandemic (H1N1) 2009 compared with cases in the general population (*9*), but the risk for infection for healthcare workers, and between different subgroups of healthcare workers, remains unclear (*9*).

During the initial epidemic wave of pandemic (H1N1) 2009 in Singapore, June–September 2009, we conducted a prospective seroepidemiologic cohort study among healthcare workers in Tan Tock Seng Hospital (TTSH), Singapore, by using serial blood specimens to determine antibody levels against pandemic (H1N1) 2009 as a marker of serologic infection. We describe the incidence of serologic evidence of infection and associated occupational and nonoccupational risk factors for infection in this cohort of healthcare workers.

## Methods

### Study Setting

TTSH is an acute-care hospital in Singapore with 1,100 beds and ≈6,000 healthcare workers; it has a designated center, the Communicable Disease Centre, for management of outbreaks of emerging infections. Following the activation of Singapore’s pandemic response plan by the Ministry of Health on April 25, 2009, TTSH became the designated screening center and isolation facility for all adult case-patients with pandemic (H1N1) 2009, although the first case-patient with the infection in Singapore did not receive a diagnosis and was not admitted to the hospital until May 26, 2009 (*12*). Intensive surveillance and testing of staff who had acute respiratory illness (ARI) symptoms confirmed the first case of pandemic (H1N1) 2009 in a TTSH staff member 4 weeks later, on June 22, 2009, several days after sustained community transmission had occurred in Singapore (*13**,**14*).

### Study Design

This study was part of a larger seroepidemiologic investigation involving 3 other cohorts in Singapore: community-dwelling adults, military personnel, and staff and residents of 2 long-term care facilities (*15*). In TTSH healthcare workers (as well as in the community-dwelling adults and military personnel), up to 3 serial serum samples were taken from each person. The samples included 1) a baseline sample collected during June 22–July 7, 2009, before widespread local transmission of pandemic (H1N1) 2009; 2) an intraepidemic follow-up sample, collected during August 19–September 3, 2009, ≈4 weeks after pandemic (H1N1) 2009 epidemic activity had peaked in Singapore; and 3) a postepidemic follow-up sample, collected during September 29–October 15, 2009, >4 weeks after epidemic activity subsided in late August 2009 (*14*).

In addition, we used standardized self-administered questionnaires to obtain baseline demographic information, seasonal influenza vaccination status, and household composition data at the time of baseline sample collection. Symptoms and possible exposures in the intervening periods between samples were elicited through follow-up questionnaires administered at the time the intraepidemic and postepidemic samples were taken. Symptom reviews covered episodes of ARI, defined as a new onset illness with any respiratory symptoms (rhinorrhea, nasal congestion, sore throat, or cough), with febrile respiratory illness (FRI) being an ARI episode with self-reported fever or a body temperature (where available) >37.5°C. Information on symptomatic episodes was augmented through sickness absenteeism rates and staff medical records for details such as dates of illness and tests to confirm pandemic (H1N1) 2009 infection. Exposure data covered nonoccupational exposures such as travel out of Singapore and episodes of ARI and FRI in household members, as well as occupational exposures such as care of patients with confirmed pandemic (H1N1) 2009 infection and contact with sick colleagues who have subsequently confirmed pandemic (H1N1) 2009. Healthcare workers were also asked, when appropriate, how often they used either a surgical mask or N95 respirator during patient care and to estimate their average daily number of visitor and patient contacts. The number of colleagues in the same work area was used as a proxy indicator of the number of staff-to-staff contacts.

### Recruitment of Study Participants

For the purposes of our study, we defined healthcare workers as any full-time staff personnel employed by TTSH, regardless of the nature of their work. We used internal hospital email systems and word-of-mouth referrals to invite all personnel >21 years of age to participate. In addition, mobile teams were sent to appropriate hospital locations such as wards, outpatient clinics, and other major work areas, such as operating theater, radiology, laboratory medicine, pharmacy, physiotherapy, and occupational therapy departments. Included in these were 3 postulated high-exposure settings: the designated isolation wards for patients with pandemic (H1N1) 2009, the emergency department through which patients with pandemic (H1N1) 2009 were being admitted, and the medical intensive care and high-dependency units where patients with the most severe pandemic (H1N1) 2009 infections were treated. Healthcare workers with ARI episodes that occurred within the 2 weeks before baseline samples were obtained were excluded, given that enrollment stopped 2 weeks after the first TTSH staff member received a diagnosis of pandemic (H1N1) 2009. Written informed consent was obtained for all participants. The study was approved by the ethics review boards of the National Healthcare Group.

### Laboratory Methods and Computation of Geometric Mean Titer

Samples were tested by hemagglutination inhibition (HI) assays following standard protocols at the World Health Organization Collaborating Centre for Reference and Research on Influenza in Melbourne, Australia (*16*). Serum samples were pretreated with receptor-destroying enzyme II (Deka Seiken Co. Ltd., Tokyo, Japan), 1:4 (vol/vol), at 37°C for 16 h before enzyme inactivation by the addition of an equal volume of 1.6% trisodium citrate (Ajax Chemicals, Melbourne, Victoria, Australia) and incubation at 56°C for 30 min. A/California/7/2009 A(H1N1) pandemic virus was purified on a sucrose gradient, concentrated, and inactivated with β-propiolactone, to create an influenza zonal pool preparation. Twenty-five microliters (4 hemagglutination units) of influenza zonal pool A/California/7/2009 virus were incubated at room temperature with an equal volume of receptor-destroying enzyme II–treated serum samples, with different wells for serum titrated in 2-fold dilutions from 1:10 to 1:1,280 in phosphate-buffered saline. After incubation of serum for 1 h, 25 μL of 1% (vol/vol) turkey erythrocytes was added to each well. HI was read after 30 min, with titers expressed as the reciprocal of the highest dilution of serum in which hemagglutination was prevented. For computing geometric mean titers (GMTs), we assigned titers <10 a value of 5, and titers ≥1,280 a value of 1,280. These values were then log transformed before we computed means and associated 95% confidence intervals (CIs). GMTs were then obtained by back transformation (*17*).

The HI assay was assessed on paired serum samples from 56 case-patients with pandemic (H1N1) 2009 confirmed by reverse transcription–PCR. The assay had a sensitivity of 80% when seroconversion was defined as a ≥4-fold increase in antibody titers between the first and second blood specimens (*15*).

### Sample Size Calculations and Outcomes of Interest

We targeted a final sample size of at least 500, which would have given a power of 90% to detect (with a 2-sided p<0.05) seroconversion rates that were 10% higher for the healthcare workers cohort than the concurrently taken community sample, which was assumed would have seroconversion rates of 25% (on the basis of the 1957 pandemic) (*18*). The target sample size would also have given a power of >70% to detect a ≥2× risk of seroconversion in a healthcare worker subgroup of ≈100 than in the rest of the healthcare worker population, assuming overall seroconversion risk in healthcare workers exceeded 10%.

The primary outcome of interest was seroconversion, which was defined as a ≥4-fold increase in antibody titers between any successive pair of blood specimens. We performed univariate and multivariate logistic regression with demographic information, seasonal influenza vaccine status, titers in the baseline sample, occupational and nonoccupational related exposures to assess their contribution to seroconversion, with results presented as odds ratios (ORs) with asymptotic Wald 95% CI and 2-sided p values. Multivariate analysis involved backward stepwise logistic regression with all variables significant at p<0.10; only variables which improved model fit at p<0.10 were included in the final model. Where appropriate, 95% CIs were also presented, along with χ^2^ and Student unpaired *t* test results for differences between proportions and means, respectively. All statistical analyses were performed by using STATA 10.0 (StataCorp, College Station, TX, USA).

## Results

We enrolled a total of 558 healthcare workers into the study, of which 96% (537/558) had ≥1 follow-up blood sample; 6 participants were excluded because of missing follow-up review questionnaires, leaving 531 persons for analysis. Of these, 35 (6.6%) seroconverted. Table 1 compares selected characteristics of seroconverters and nonseroconverters. Seroconverters were sampled earlier than nonseroconverters (49% vs. 38% in the first week of enrollment), and 86% of seroconverters had both follow-up samples compared with 81% of nonseroconverters, but these differences were not significant (p = 0.20 and p = 0.73, respectively). Seroconverters were slightly more likely to have received seasonal influenza vaccine than were nonseroconverters (97% vs. 91%), but this difference was not significant. There were no also significant differences between seroconverters and nonseroconverters by age or gender. HI titers in baseline samples from nonseroconverters were higher than in baseline samples from seroconverters (GMT 7.8 vs. 5.9; p = 0.02). Among seroconverters, 63% and 51% reported having an ARI and FRI episode, respectively, and only 15% and 8% of nonseroconverters reported having an ARI and FRI episode, respectively (p<0.01 for both).

**Table 1:** Selected characteristics of healthcare workers by seroconversion status for pandemic (H1N1) 2009, Singapore, 2009*

Characteristic	No. (%) seroconverters, n = 35	No. (%) nonseroconverters, n = 496	p value
Baseline sample timing			0.20†
Jun 22–26	17 (49)	187 (38)	
Jun 28–Jul 7	18 (51)	309 (62)	
Follow-up samples taken			0.73†
Intraepidemic only	3 (9)	65 (13)	
Postepidemic only	2 (6)	31 (6)	
Intraepidemic and postepidemic	30 (86)	400 (81)	
Female	30 (86)	411 (83)	0.66†
Seasonal influenza vaccination	34 (97)	449 (91)	0.19†
ARI episode‡	22 (63)	75 (15)	<0.01†
FRI episode‡	18 (51)	41 (8)	<0.01†
Age, y, mean (95% CI)	35 (31–39)	34 (33–35)	0.76§
GMT for baseline sample (95% CI)	5.9 (5.3–6.5)	7.8 (7.3–8.3)	0.02§

*ARI, acute respiratory illness; FRI, febrile respiratory illness; CI, confidence interval; GMT, geometric mean titer. †χ^2^ test comparing seroconverters and nonseroconverters. ‡Healthcare workers who seroconverted are considered to have had an ARI or FRI episode if the date of onset preseded the date when seroconversion was detected. §Student *t* test comparing seroconverters and nonseroconverters.

Most of our participants were nurses (290/531, 55%; Table 2); allied health staff, which included mostly participants from paramedical professions such as pharmacists, laboratory medicine technicians, physiotherapists and occupational therapists, formed the second largest group (116/531, 22%); ancillary and support staff, which included mainly hospital attendants and patient service associates, formed the next largest group (69/531, 13%); and administrative support staff (35/531, 7%) and doctors (21/531, 4%) made up the rest. Seroconversion rates were highest in nurses (28/290, 10%) and lowest in allied health staff (2/116, 2%). To facilitate interpretation, allied health staff were designated the reference group for computing ORs; only nurses had a significantly higher odds of infection compared with allied health staff (OR 6.1, 95% CI 1.4–26.0; p = 0.02). Compared with those working in non–patient care areas, participants whose primary work area was an inpatient ward had higher odds for seroconversion (OR 1.4, 95% CI 0.5–3.5; p = 0.54), while those in other patient care settings had lower odds for seroconversion (OR 0.5, 95% CI 0.2–1.5; p = 0.21), but neither result was significant. Significantly higher odds for seroconversion were also observed for participants whose primary work area was in pandemic (H1N1) 2009 isolation wards (OR 4.8, 95% CI 1.5–15.6; p<0.01).

**Table 2:** Univariate analysis of occupational risk factors for pandemic (H1N1) 2009 for 531 healthcare workers, Singapore, 2009*

Risk factor	No. participants	No. (%) seroconverted	Crude OR (95% CI)	p value
Occupational subgroup				
Allied health	116	2 (2)	Referent	
Nurses	290	28 (10)	6.1 (1.4–26.0)	0.02
Ancilllary and support	69	2 (3)	1.7 (0.2–12.4)	0.60
Administration	35	2 (6)	3.5 (0.5–25.5)	0.22
Doctors	21	1 (5)	2.9 (0.2–32.9)	0.40
Direct patient contact				
No	71	4 (6)	Referent	
Yes	460	31 (7)	1.2 (0.4–3.5)	0.73
Primary work area				
Nonpatient care areas	83	6 (7)	Referent	
Inpatient wards	210	20 (10)	1.4 (0.5–3.5)	0.54
Other patient care settings†	238	9 (4)	0.5 (0.2–1.5)	0.21
Work in high exposure settings				
Pandemic (H1N1) 2009 isolation wards	514	31 (6)	Referent	
No	514	31 (6)	Referent	
Yes	17	4 (24)	4.8 (1.5–15.6)	<0.01
Emergency department	507	33 (7)	Referent	
No	507	33 (7)	Referent	
Yes	24	2 (8)	1.3 (0.3–5.8)	0.73
Medical ICU/HDU				
No	514	33 (6)	Referent	
Yes	17	2 (12)	1.9 (0.4–8.9)	0.39
Contact with patient who had pandemic (H1N1) 2009				
No	409	23 (6)	Referent	
Yes	122	12 (10)	1.8 (0.9–3.8)	0.10
Contact with sick colleague(s) who had pandemic (H1N1) 2009				
No	484	28 (6)	Referent	
Yes	47	7 (15)	2.9 (1.2–6.9)	0.02

*OR, odds ratio; CI, confidence interval; ICU, intensive care unit, HDU, high-dependency unit. †Includes allied health and medical staff from departments with both inpatient and outpatient coverage and staff from all other noninpatient departments involved in patient care.

Participants who had contact with patients who had pandemic (H1N1) 2009 had marginally but not significantly increased odds of seroconversion (OR 1.8, 95% CI 0.9–3.7; p = 0.10). Those who reported having contact with a sick colleague(s) whose illness was subsequently diagnosed as pandemic (H1N1) 2009 had significantly increased odds of seroconversion (OR 2.9, 95% CI 1.2–6.9; p = 0.02).

Results of the univariate analysis for nonoccupational exposures are presented in the Figure. Healthcare workers from larger households had increased odds of seroconversion (OR 1.2 per additional household member, 95% CI 1.0–1.4; p = 0.04), but no discernible association was seen between seroconversion and having another healthcare worker in the same household or reporting another household member with ARI or FRI symptoms during the study. However, having a child or adolescent in the household increased the odds of seroconversion. In particular, significantly higher ORs were observed if the healthcare workers reported a child 5–12 years of age in the household (OR 2.1, 95% CI 1.0–4.4; p = 0.05), with the ORs being even higher if a child 5–12 years of age in the household had FRI symptoms (OR 4.1, 95% CI 1.1–15.6; p = 0.04).

**Figure:**
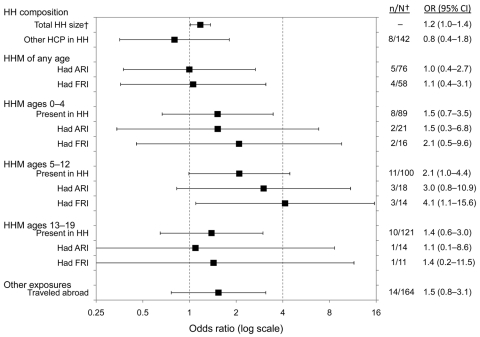
Univariate analysis for nonoccupational exposures to pandemic (H1N1) 2009 among healthcare workers, Singapore. Error bars indicate 95% confidence intervals (CIs) for odds ratios (ORs). †n/N, no. of seroconverters/no. in strata. HH, household; HCP, healthcare provider; HHM, household member; ARI, acute respiratory illness; FRI, febrile respiratory illness.

The variables included in the final multivariate analysis are shown in Table 3. Being a nurse remained significantly associated with increased odds of infection (OR 4.5, 95% CI 1.0–19.6; p = 0.05), as did having pandemic (H1N1) 2009 isolation wards as a primary work area (OR 4.5, 95% CI 1.3–15.6; p = 0.02). Contact with colleagues with pandemic (H1N1) 2009 (OR 2.5, 95% CI 0.9–6.6; p = 0.06) and coming from a larger household (OR 1.2 per additional household member, 95% CI 1.0–1.4; p = 0.06) were of borderline significance. Having higher HI titers in the baseline serum sample was protective (OR 0.5 per unit of increase, 95% CI 0.3–1.0; p = 0.05).

**Table 3:** Multivariate analysis of risk factors associated with seroconversion for pandemic (H1N1) 2009 in 531 healthcare workers, Singapore, 2009*

Risk factor	Adjusted OR (95% CI)	p value
Occupational subgroup		
Allied health	Referent	
Nurses	4.5 (1.0–19.6)	0.05
Ancilllary and support	1.5 (0.2–11.1)	0.69
Administration	3.6 (0.3–42.8)	0.31
Doctors	3.8 (0.5–28.7)	0.19
Pandemic (H1N1) 2009 isolation wards vs. all others	4.5 (1.3–15.6)	0.02
Contact with colleague(s) who had pandemic (H1N1) 2009 vs. none	2.5 (0.9–6.6)	0.06
Household size (per additional household member)	1.2 (1.0–1.4)	0.06
HI titer in baseline sample (per unit of increase)†	0.5 (0.3–1.0)	0.05

*OR, odds ratio; CI, confidence interval; HI, hemagglutination inhibition. †For every unit increase in baseline (sample A) titer, where the integer values of 0–8 denote titers <0, 10, 20, 40, 80, 160, 320, 640, and >1,280, respectively.

In Table 4, allied health participants are compared with nurses, the groups with the lowest and highest seroconversion rates respectively; the latter was further stratified by whether they worked in inpatient wards (ward based vs. non–ward based). The proportion who seroconverted was slightly, but not significantly, higher in ward-based nurses than in non–ward-based nurses (11% vs. 8%; p = 0.53). Ward-based nurses were from significantly larger households than the other 2 groups (p<0.01 vs. allied health, p = 0.01 vs. non–ward-based nurses). No significant difference was found in the proportion who reported using face masks all or almost all of the time in patient care, but ward-based nurses were significantly more likely to have had seasonal influenza vaccine than were allied health workers (p<0.01). Key differences were found in the mean number of contacts and occupational related factors. Non–ward-based nurses mostly worked in large areas, including operating theaters, the emergency department, and outpatient clinics, and hence had significantly higher numbers of contacts than either ward-based nurses or allied health workers (p<0.01 on all measures). On the other hand, ward-based nurses were significantly more likely to be in contact with patients with confirmed pandemic (H1N1) 2009 (p<0.01 vs. non–ward-based and allied health), and allied health staff were significantly less likely to be in contact with a sick colleague who had pandemic (H1N1) 2009 (p<0.01 vs. either nursing group).

**Table 4:** Comparison of risk factors among allied health staff, ward-based nurses, and non–ward-based nurses for exposures to pandemic (H1N1) 2009, mask use, and work-related contacts, Singapore, 2009*

Risk factor	1: Allied health staff, n = 116	2: Non–ward-based nurses, n = 103	3: Ward-based nurses, n = 187	p values†		
				2 vs. 1	3 vs. 1	3 vs. 2
Seroconverted in study period, %	2 (0–6)	8 (4–15)	11 (7–16)	0.05	<0.01	0.53
Mean age, y	32 (30–33)	34 (32–36)	32 (31–34)	0.14	0.81	0.21
Mean household size	4.8 (4.4–5.1)	4.8 (4.4–5.2)	5.5 (5.1–5.8)	0.96	<0.01	0.01
Household members with FRI in the following age groups, %						
0– 4 y	3 (1–7)	3 (1–8)	2 (1–5)	1.00	1.00	0.70
5–12 y	2 (0–6)	4 (2–10)	1 (0–4)	0.42	0.64	0.19
13–19 y	1 (0–5)	3 (1–8)	2 (1–5)	0.34	0.65	0.70
Masks for patient care all or almost all the time, %	64 (53–74)	71 (59–80)	69 (61–77)	0.48	0.54	0.87
Valid responses‡	76	69	130			
Received seasonal influenza vaccine, %	84 (77–90)	91 (84–95)	96 (92–98)	0.15	<0.01	0.19
Geometric mean no. colleagues in work area	27 (24–31)	48 (39–59)	23 (20–25)	<0.01	0.05	<0.01
Valid responses‡	101	89	147			
Geometric mean no. patient contacts per day	15 (12–18)	37 (26–52)	19 (17–22)	<0.01	0.03	<0.01
Valid responses‡	73	59	127			
Geometric mean no. visitor contacts per day	12 (9–15)	28 (21–38)	15 (13–18)	<0.01	0.04	<0.01
Valid responses‡	75	58	133			
Occupational-related exposures, %						
Direct patient contact	84 (77–90)	91 (84–95)	99 (97–100)	0.15	<0.01	<0.01
Contact with patients who had pandemic (H1N1) 2009	14 (9–21)	19 (13–28)	41 (34–48)	0.28	<0.01	<0.01
Contact with colleague(s) who had pandemic (H1N1) 2009	2 (0–6)	15 (9–23)	14 (10–20)	<0.01	<0.01	0.86

*Values in parentheses are 95% confidence intervals. FRI, febrile respiratory illness. †p values by Fisher exact test for proportions and unpaired Student *t* test for means. ‡Based on participants who answered this questionnaire item; all other analyses are based on no. participants in that occupational subgroup.

## Discussion

In this study, we used paired serum samples to assess infection rates and risk factors for infection in healthcare personnel during an influenza pandemic in an acute care hospital in Singapore. We observed surprisingly lower seroconversion rates in healthcare personnel than in the rest of the community, as was emphasized in another publication (*15*), and found that a mixture of occupational and nonoccupational exposures were associated with risk for infection.

When the study was planned, we had expected the healthcare workers cohort to have a higher seroincidence than a group of community-dwelling adults, given previous reports of pandemic and nonpandemic influenza outbreaks in hospitals (*19**,**20*), our own experience with SARS (*1*), and recent work showing the intensity of work-related contacts in the healthcare setting (*3*). Instead, we found that only 7% of our healthcare workers seroconverted, compared with 13% of participants in the community cohort (*15*). Our study corroborates case-reporting data in the United States, which suggest that healthcare workers did not have a higher incidence of infection than the general community, without being subject to biases that might arise from underreporting or differential case ascertainment (*9*).

Although definitively attributing the low infection rates in healthcare workers to improved infection control practices is difficult without the appropriate control groups, much evidence supports the efficacy of the common bundle of measures used in hospitals to reduce spread of respiratory viruses (*21*). Notably, there was a high level of preparedness and widespread implementation of airborne and respiratory droplet precautions and other pandemic (H1N1) 2009 infection control practices in healthcare institutions in the United States, Singapore, and elsewhere (*11**,**13**,**22**–**24*).

However, our study also suggests that the risk to healthcare staff should not be underestimated. We found some occupational factors associated with seroconversion. The higher seroconversion rates in nurses posted to designated pandemic (H1N1) 2009 isolation wards should be interpreted with some caution in view of the small number of seroconversion events (2 of those infected had symptoms and 2 did not) and participants (n = 17) from these wards. Since masks (either surgical or N95 masks) were widely used in all clinical areas around the hospital, this group essentially had the same level of protection as other staff while being far more intensely exposed to pandemic (H1N1) 2009. The higher risk for seroconversion for nurses on multivariate analysis also deserves notice. Nurses had higher seasonal influenza vaccination rates and were more compliant than other occupational subgroups in following preventive measures such as mask use. However, they also were more likely to be exposed to patients as well as to have colleagues with confirmed pandemic (H1N1) 2009; the latter factor was significantly associated with seroconversion by univariate analysis (and of borderline significance on multivariate analysis), and staff-to-staff transmission was also implicated in TTSH and elsewhere (*10**,**13*). Non–ward-based nurses also had higher contact rates than the other main occupational subgroup (allied health staff), a factor that we could not account for in multivariate analysis (as questions on contact rates were not answered by all participants). We also could not account for the nature of patient contacts, which might be more prolonged and intense in nurses (*3*). We suggest that our finding of the higher seroconversion risk in nurses is the result of residual confounding by the sum of these factors, many of which are an integral part of the nursing profession.

Lastly, our study suggests that nonoccupational exposures should not be forgotten as a potential source of healthcare worker infections. Other studies based on case investigations have also attributed some infections to community sources, and in our study, we found that having a child of primary school age was a risk factor on univariate analysis, particularly if that child had an FRI during the study period, although the direction of transmission in the latter could not be ascertained. Studies on nonpandemic influenza have found that index cases from pediatric age groups were more likely to generate secondary cases (*25**,**26*), although the same was not observed with pandemic (H1N1) 2009 (*27*). The effect of having children in the household was superseded in multivariate analysis by overall household size, which was unsurprising since households with children also tended to be larger. In any case, the significance of such nonoccupational exposures should be taken into account in any hospital-level pandemic preparedness plan.

We do acknowledge several limitations in our study. First, our findings are based on data from healthcare workers from just 1 hospital. Moreover, the unexpectedly low seroconversion rates in our cohort reduced the power of the study to investigate exposures more weakly associated with the outcome. We were also unable to assess the usefulness of personal protective equipment due to the lack of appropriate control groups. The resolution of exposure data from what was a self-administered questionnaire survey was also lower than insights that may be gained from detailed case investigations or exposure diaries that have been used in the healthcare setting (*3**,**11*). Finally, the lack of randomization also leaves scope for bias in our results.

An effective vaccine for pandemic (H1N1) 2009 has now been introduced, and this will likely reduce intrahospital risk of infection from this particular strain of influenza until significant genetic drift occurs, provided healthcare institutions can overcome the challenges to achieving high vaccine coverage rates in healthcare personnel (*28**,**29*). Seasonal influenza vaccination rates may be atypically high in TTSH because of its designated status as a first-line screening and referral center; 1 study on healthcare workers from 2 other hospitals in Singapore found that only 39% of participants were vaccinated (*30*). Although the low incidence of healthcare workers infections provides some suggestion that measures in place during the pandemic were effective, our findings suggest that some occupation-related risk factors remain. Nurses, particularly those working in pandemic (H1N1) 2009 isolation wards, were disproportionately affected, possibly because their higher levels of protective behaviors inadequately compensated for their increased occupational risk. This situation should be recognized when planning for future pandemics.
